# Electricity as a Means of Resuscitation

**Published:** 1873-06

**Authors:** 


### ARTICLE XII.

### *Electricity as a Means of Resuscitation.*

Allan McLane Hamilton, M. D., New York, in a paper on this subject (*American Practitioner*,) speaks of the following results arrived at from his own practice and experiments:

1. That it is useless to expect good results if five minutes have elapsed since life appears extinct.

2. That the current should be applied faithfully and steadily, one pole being placed on the ensiform cartilage, the other on the base of the skull, or over the track of the great nerves of the neck.

3. That the faradic and interrupted galvanic currents are the best.

4. That the current should be applied some time after perspiratory movements have become regular.

In conclusion the author says:

"The necessity of having a battery within reach is apparent. Every practitioner should have a small one for emergencies. They should be kept at each life-saving station on the coast, ready charged, with directions for immediate use. If this were done, he doubts if the per centage of deaths would be so great as it now is. Artificial respiration by the production of muscular movements is a very valuable means of restoration ; but a force that acts directly upon the nerves supplying the muscles of respiration, is by far the surest and best."—*Medical Examiner.*

---

